# Primary dendrites of mitral cells synapse unto neighboring glomeruli independent of their odorant receptor identity

**DOI:** 10.1038/s42003-018-0252-y

**Published:** 2019-01-08

**Authors:** Hirofumi Nishizumi, Akihiro Miyashita, Nobuko Inoue, Kasumi Inokuchi, Mari Aoki, Hitoshi Sakano

**Affiliations:** 1 Department of Biophysics and Biochemistry, Graduate School of Science, The University of Tokyo, 2-11-16 Yayoi, Bunkyo-ku, Tokyo, 113-0032 Japan; 2 Department of Brain Function, School of Medical Sciences, University of Fukui, 23-3 Shimo-aizuki, Matsuoka, Fukui 910-1193 Japan

## Abstract

In the mouse olfactory bulb, neural map topography is largely established by axon–axon interactions of olfactory sensory neurons (OSNs). However, to make the map functional, the OSNs must make proper connections to second-order neurons, the mitral cells. How do the mitral-cell dendrites find their partner glomeruli for synapse formation with OSN axons? Here, we analyze dendrite connections of mitral cells in various mutant mice in which glomerular formation is perturbed. Our present results support the proximity model, whereby mitral cells tend to connect primary dendrites to the nearest neighboring glomeruli regardless of their odorant receptor identities. The physical location of glomeruli rather than the odorant-receptor specificity appears to play a key role in matching mitral cells with their partner OSN axons.

## Abstract (web summary)

Hirofumi Nishizumi et al. examined dendrite connections in olfactory bulb mitral cells of mice with perturbed glomerular formation. Their findings support the model that mitral cells connect primary dendrites to neighboring glomeruli.

## Introduction

In the mouse olfactory system, various odorants are detected using a repertoire of approximately 1000 odorant receptors^1^. Olfactory sensory neurons (OSNs) in the olfactory epithelium stochastically express only one functional odorant receptor gene in a monoallelic manner^2–4^. Furthermore, OSNs expressing the same odorant receptor species converge their axons to a specific site to form a glomerular structure. Thus, each glomerulus represents one odorant receptor species in the olfactory bulb^5–7^. In mice, odorous information detected in the olfactory epithelium is converted to a two-dimensional map of activated glomeruli in the olfactory bulb, enabling the brain to discriminate a variety of odorants^8^.

In the mouse olfactory bulb, the odorous information is further processed by local neuronal circuits and conveyed by mitral/tufted (M/T) cells to the olfactory cortex^9^. In the olfactory systems of the fly and nematode, projection neurons are pre-specified by the cell lineage and birth order to form synapses with incoming axons of olfactory receptor neurons (ORNs)^10–13^. This genetically-programmed pre-specification of ORNs generates hard-wired circuits that induce stereotyped innate odor responses. In contrast, in the mouse olfactory system, much of targeting occurs autonomously by axon–axon interactions of OSNs without involving target cues^14–17^. Even in mice, however, proper matching and connections are required to induce innate odor responses^18–20^. Then, how are mouse M/T cells able to find their partner glomeruli for synapse formation?

Here, we study matching between the OSN axons and mitral-cell dendrites in the mouse olfactory system. The question to be answered is how both parties are able to find the right counterparts. One possibility is that OSN axons and mitral-cell dendrites recognize the partners’ identity when the matching is taking place. If this is the case, the identity of OSNs is likely established by the expressed odorant receptor species. This then engenders the question of the identity of mitral cells and how it is recognized by OSN axons. Is there any molecular code expressed in the mitral-cell dendrites for finding their partner glomeruli? Another possibility is that there is no such a molecular code of mitral cells to be recognized by OSN axons for proper matching to trigger the synapse formation. Mitral-cell dendrites may find their partner OSN axons based on their proximity to the target glomeruli without regard to odorant-receptor specificity. If this is the case, it is important for mitral cells to migrate to proper locations in the olfactory bulb to make the circuit functional^19^. In order to address what mediates the matching with glomeruli, we analyze partner finding and dendrite selection of mitral cells in various mutant mice with deficits in glomerular map formation.

## Results

### Dendrite selection and odorant receptor identities of glomeruli

To study dendrite maturation of mitral cells, the transgenic (Tg) mouse pThy1-YFP^21^ was used to selectively visualize mitral cells in which the Thy1 promoter specifically induces expression of yellow fluorescent protein (YFP). Two-photon laser microscopy enabled us to analyze three-dimensional (3D) images of whole mitral-cell dendrites. On postnatal day 1 (P1), mitral cells extend multiple dendrites toward the glomerular layer, interacting with neighboring glomeruli (Supplementary Fig. 1a). At later stages, only one dendrite is selected as a primary dendrite, and branches are removed by pruning. As a result, each mitral cell forms a specific synapse with a single glomerulus^22^ (Supplementary Fig. 1a and b). To examine whether mitral cells find the partner glomeruli on the basis of their odorant-receptor specificity for dendrite selection, we conducted the following experiment.

**Fig. 1:**
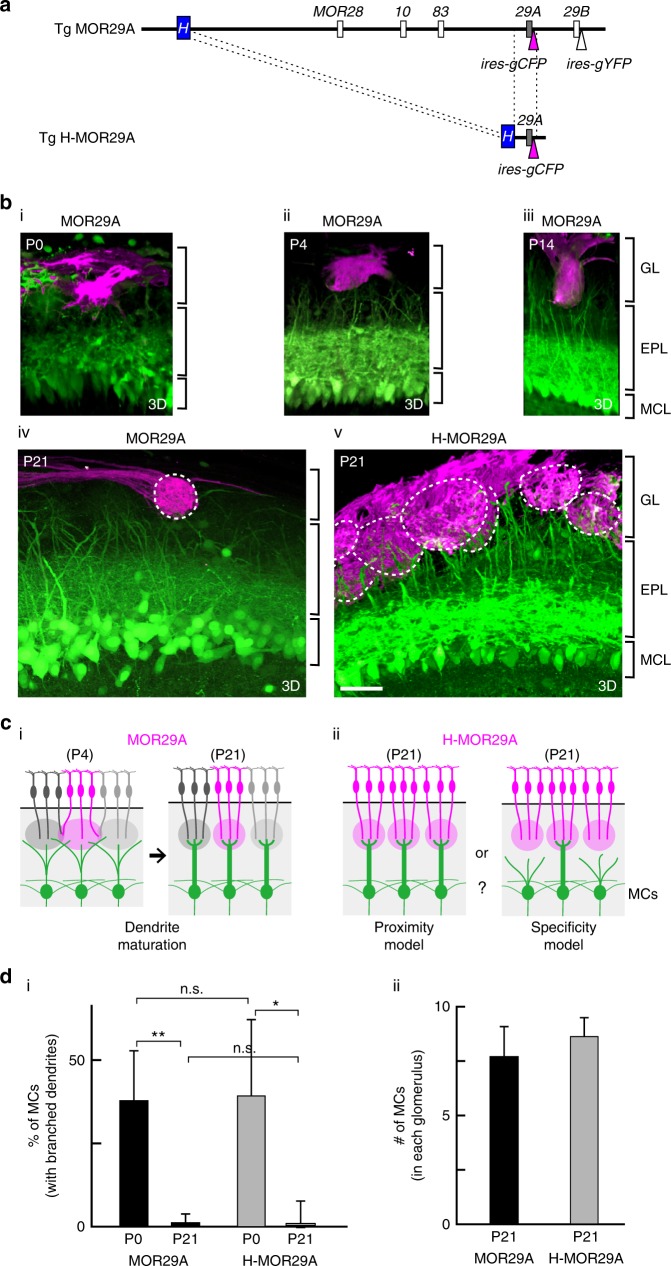
Dendrite extension of mitral cells (MCs) to the multiple glomeruli with the same odorant receptor identity. **a** Schematic diagrams of the transgenic (Tg) constructs. The *Tg MOR29A* contains 223 kb DNA region of the *MOR28* cluster, where *MOR29A* and *MOR29B* genes are tagged with *ires-gapECFP* and *ires-gapEYFP*, respectively, in the bacterial artificial chromosome (BAC). In the *Tg H-MOR29A*, the *H* enhancer is attached to the *MOR29A* minigene tagged with *ires-gapECFP*. **b** Dendrite maturation of MCs expressing the *Tg MOR29A*. Olfactory bulb (OB) sections of Tg MOR29A mice were analyzed at P0, P4, and P14 (i, ii, iii). Dendrite morphologies of the MCs are compared between the Tg MOR29A and Tg H-MOR29A mice at P21 (iv, v). MCs are selectively visualized by YFP in the Tg pThy1-YFP mouse. OSN axons expressing MOR29A and MCs are colored in red and green, respectively. GL, glomerular layer; EPL, external plexiform layer; MCL, mitral cell layer. MOR29A glomeruli are encircled by dotted lines. Scale bar, 50 μm. Three dimensioned (3D) videos are available in the supplementary information. **c** Schematic drawings of glomerular matching of MCs. Primary dendrites are selected in MCs after matching with partner glomeruli (i). In the H-MOR29A mouse, glomeruli with the same odorant-receptor specificity, Tg-MOR29A, are clustered in the OB region where the glomerulus for the endogenous MOR29A is located (ii). Does matching take place based on the odorant-receptor specificity of glomeruli or physical distance to the partner? **d** The ratios of MCs (%) retaining branched dendrites are compared between the Tg MOR29A and Tg H-MOR29A mice at P0 and P21 (i). *n* = 16 MCs for H-MOR29A and *n* = 40 MCs for MOR29A. n.s., *p* > 0.05; *0.05 > *p* > 0.01; **p < 0.01; Welch’s *t*-test; error bar, 95% C.I. The numbers of MCs connecting to each MOR29A glomerular structure is also compared between the Tg MOR29A and Tg H-MOR29A mice (ii)

Using the Tg H-odorant receptor system^23,24^, we generated a situation where multiple glomeruli with the same odorant receptor identity are clustered in a restricted area of the olfactory bulb (Fig. 1). We analyzed mitral-cell dendrites in the mouse line, Tg H-MOR29A made from the Tg MOR29A mouse^25^. In the *Tg H-MOR29A* construct, the *H* element^23,24^ that enhances the gene choice was attached to the *Tg MOR29A* minigene tagged with *ires-gapECFP* for fluorescent visualization of Tg MOR29A-positive glomeruli (Fig. 1a). Usually, a single glomerulus is formed for each odorant receptor species, as seen for MOR29A in the Tg MOR29A mouse (Fig. 1b and Supplementary movies 1-4). However, in the Tg H-MOR29A mouse, a cluster of glomeruli with the same odorant-receptor specificity was formed for MOR29A in the dorsal region of the olfactory bulb (Fig. 1b and Supplementary movie 5). This occurred because the frequency of the choice for the *Tg MOR29A* is markedly increased by the addition of the *H* enhancer^23,24^. It was expected that if partner matching of mitral cells occurs on the basis of odorant-receptor specificity of glomeruli, only one MOR29A-positive glomerulus located at the original glomerular site for the endogenous MOR29A would receive primary dendrites from mitral cells underneath (Fig. 1c, specificity model). All other MOR29-positive ectopic glomeruli would not receive primary dendrites from the mitral cell layer, because these mitral cells were supposed to be paired with glomeruli having different odorant receptor identities from MOR29A (Fig. 1c, specificity model).

To examine whether this odorant receptor-specificity model is correct for partner matching of mitral cells, we analyzed 3D images of MOR29A glomeruli using a two-photon laser microscope in 3-week-old Tg H-MOR29A mice. No abnormality or impairment of dendrite maturation/selection was seen near the MOR29A glomeruli: we did not find mitral cells extending multiple or branched dendrites to more than one Tg MOR29A glomeruli (Fig. 1d, i). A similar result was obtained at earlier stages, P7 (Supplementary Fig. 1c). Since mitral cells with branched dendrites could have been eliminated during development, we counted the number of mitral cells connecting to each MOR29A glomerulus in both Tg H-MOR29A and Tg MOR29A mice. No decrease in the number of mitral cells was found for the MOR29A glomeruli labeled with enhanced cyan fluorescent protein (ECFP) in the Tg H-MOR29A mouse compared with the Tg MOR29A mouse (Fig. 1d, ii). As the ectopic MOR29A glomeruli in the Tg H-MOR29A mouse are accepting the primary dendrites normally as in the control, it appears that mitral cells are not selecting the partner glomeruli based on the odorant-receptor specificity. It is likely that a mitral cell is such a cell-type that selects one primary dendrite for a nearby glomerulus (Fig. 1c, proximity model). Matching of mitral cells may be taking place based on the physical locations of partner glomeruli rather than their odorant receptor identities.

### Mitral-cell dendrites in the absence of OSN projection

**Fig. 2:**
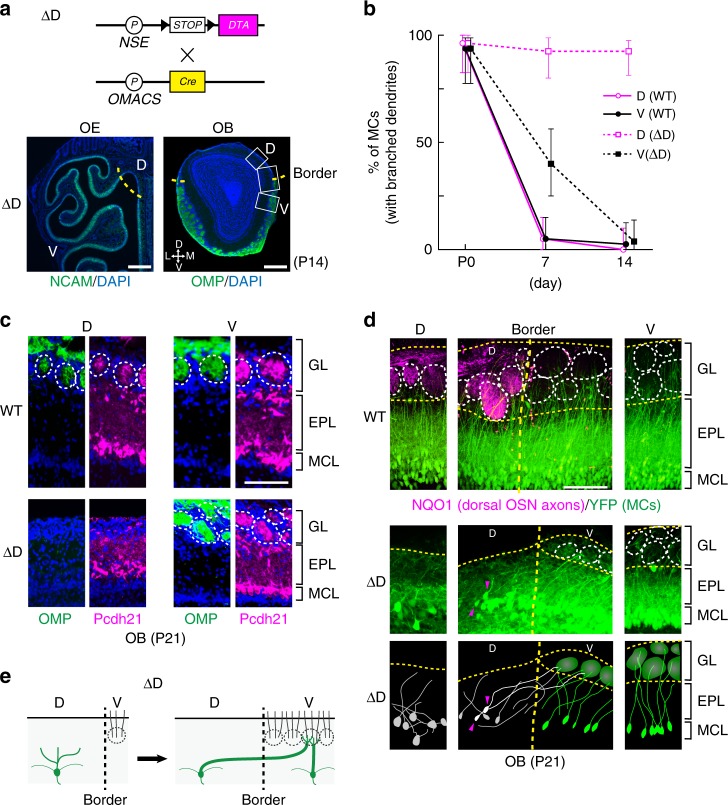
Dendrite extension of mitral cells (MCs) in the ΔD mutant. **a** Plasmid constructs for D-zone ablation (ΔD) are schematically illustrated. The *DTA* and *Cre* genes code for diphtheria toxin A and Cre recombinase, respectively. The *NSE* and *OMACS* promoters were used for region-specific expression of Cre and DTA in the olfactory epithelium (OE). The *DTA* gene is activated in the dorsal OE by the D-zone specific *OMACS* promoter, ablating D-zone OSNs. In the ΔD mutant, no NCAM signals were detected in the dorsal OE, and glomerular structures were absent in the dorsal olfactory bulb (OB). Sections were counterstained with DAPI. *n* = 3. D, dorsal; M, medial; V, ventral; L, lateral. Scale bars, 250 μm. **b** Dendrite maturation of MCs. The ratios (%) of MCs with branched dendrites are plotted for the WT and ΔD mice in the dorsal (D) and ventral (V) regions at postnatal day (P) 0, 7, and 14. **c** Detection of glomeruli in OB sections. OSN axons (green) and M/T cells (magenta) were immunostained with antibodies against OMP and Pcdh21, respectively. Note that glomerular structures are absent in the D-region OB of ΔD mice. Glomerular structures are encircled by dotted lines. GL, glomerular layer; EPL, external plexiform layer; MCL, mitral cell layer. Scale bar, 100 μm. **d** Three-dimensional stack images of mitral-cell dendrites in the WT pThy1-YFP and ΔD pThy1-YFP mice at P21. Three OB regions, dorsal (D), ventral (V), and D-V border, were analyzed. To determine the D-V border (broken lines), sections were stained with anti-NQO1 (D region marker) antibodies. Note that near the border in the ΔD, some D-side MCs (indicated by magenta arrow heads) extend long primary dendrites crossing the border to the V-side glomeruli. Schematic drawings of ΔD OB sections are shown in the lower panel. GL, glomerular layer; EPL, external plexiform layer; MCL, mitral cell layer. Scale bar, 100 μm. **e** A schematic diagram of dendrite extension of D-side MCs to the V-side glomeruli in the ΔD mice

We next studied what would happen to the mitral-cell dendrites when targeting of OSN axons is absent. To determine whether mitral cells gradually die or continue waiting for partner OSN axons, we used another mutant mouse, ∆D, where the glomeruli had been ablated in the dorsal domain of the olfactory bulb (Fig. 2a). Zone-specific expression of diphtheria toxin A (DTA) was induced from the *NSE-STOP-DTA* at the late stage of fetal development, using the dorsal-zone specific *O-MACS* promoter as a driver for the *Cre* recombinase gene^18^ (Fig. 2a). In the ∆D mice, we analyzed three different olfactory bulb regions, the dorsal, ventral, and dorsal-ventral border.

To examine the synaptic contact of OSN axons and mitral-cell dendrites, olfactory bulb sections were immunostained with antibodies against a mitral cell marker, Pcdh21^26^, and OSN axons were stained with anti-OMP antibodies. Patchy staining of Pcdh21 was detected in the OMP-positive glomerular structures in the wild-type (WT) olfactory bulb and in the ventral-region olfactory bulb of the ∆D mutant (Fig. 2c). In contrast, the Pcdh21 signals were not detected in the dorsal region of ∆D, indicating that mitral cells failed to generate tuft structures in the absence of OSN projection (Fig. 2c). In the ventral olfactory bulb of ΔD, where glomeruli were present, dendrite structures were normal, whereas in the dorsal olfactory bulb, dendrite maturation did not progress during the postnatal period (Fig. 2c). It should be noted that mitral cells do not regenerate throughout the lifetime^27^. In the ΔD mutant, mitral-cell density in the dorsal zone appeared to be normal and no sign of apoptosis was found in this region (Supplementary Fig. 2a). Interestingly, however, mitral cells stayed in the immature state and the number of branched dendrites remained the same in the dorsal zone at two weeks (2 weeks) after birth. Using the pThy1-YFP mouse, mitral cells were visualized by fluorescence and dendrite maturation was analyzed at P0, P7, and P14. In the WT control, the numbers of immature mitral cells with branched dendrites gradually decreased both in the dorsal-region and ventral-region olfactory bulbs. In contrast, in the ∆D mouse, mitral-cell dendrites stayed branched in the dorsal-region olfactory bulb where the glomeruli had been ablated (Fig. 2b). Even in the absence of OSN projection, mitral-cell dendrites retained multiple branches for at least 3 months. These observations indicate that dendrite maturation is mediated by trans-synaptic activities from OSN axons, even though some activities may come from the connecting interneurons. It appears that physical contact of mitral-cell dendrites with OSN axons is prerequisite to dendrite selection and tuft formation.

We then examined what would happen to the dendrites of mitral cells near the dorsal-ventral border in ∆D mice. If the odorant-receptor specificity or positional identity of glomeruli is not recognized by mitral cells for partner matching, mitral cells on the dorsal side of the dorsal-ventral border would not form synapse with glomeruli on the ventral side (Fig. 2e). To analyze the dendrite selection and extension of mitral cells near the border, the boundary was identified by staining glomeruli with antibodies against a dorsal-OSN marker, NQO1 (Fig. 2d). In the WT mice, mitral cells near the border extended their primary dendrites perpendicularly to the nearest glomeruli without any differences between the dorsal-region and ventral-region glomeruli (average length = 225 ± 9 μm). In contrast in the ∆D mutant, we found unusual dendrite extensions for the dorsal-region mitral cells near the border. As shown in Fig. 2d, these mitral cells extended their dendrites tangentially to the V-region glomeruli by crossing over the boundary. It is notable that some of these dendrites are longer than 400 μm (Supplementary Fig. 2b). This unexpected observation indicates that mitral cells extend primary dendrites to the nearest glomeruli, without regard to their odorant-receptor specificity or positional identity, even when they are far apart.

### Targeting of mitral-cell axons to the olfactory cortex in the absence of glomeruli

**Fig. 3:**
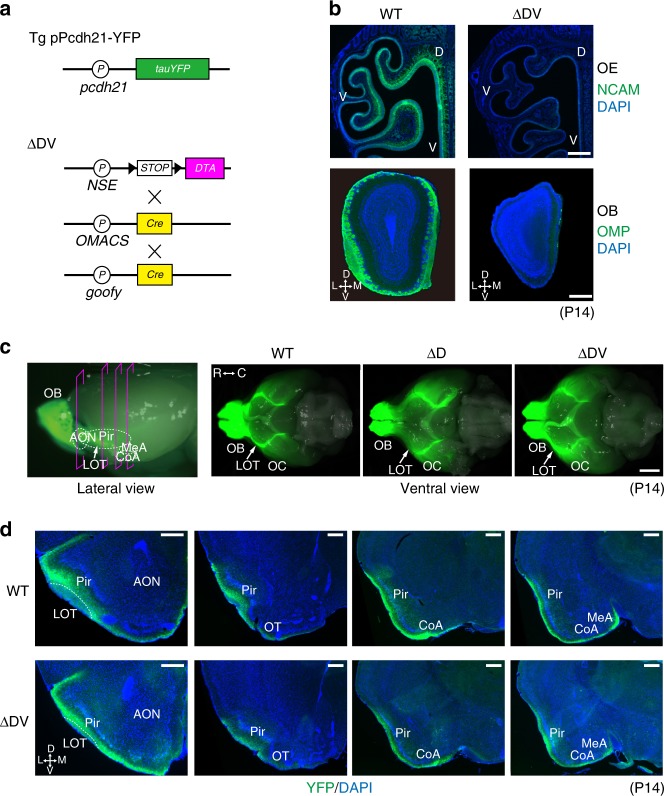
Targeting of M/T-cell axons to the olfactory cortex (OC) in the absence of OSN projection. **a** Plasmid constructs for the Tg pPcdh21-YFP and the DV-zone ablated (ΔDV) mice are schematically illustrated. The Tg pPcdh21-YFP mouse was crossed with the ΔDV mutant to visualize the M/T-cell axons using YFP. The *DTA* and *Cre* genes code for diphtheria toxin A and Cre recombinase, respectively. The *NSE*, *OMACS*, and *goofy* promoters were used for region-specific expression of Cre and DTA in the olfactory epithelium (OE). **b** Detection of OSNs and their axons. OE and olfactory bulb (OB) sections were immunostained with antibodies against NCAM and OMP, respectively. Sections were counterstained with DAPI. In the ΔDV mutant, no NCAM signals were detected in the OE, and glomerular structures were totally absent in the OB. *n* = 3. D, dorsal; M, medial; V, ventral; L, lateral. Scale bars, 250 μm. **c** M/T-cell projection to the OC. The Tg pPcdh21-YFP mouse was crossed with ΔD or ΔDV mutant so that M/T-cell axons could be visualized with YFP (green). Whole mount views of the ventral-side brain are shown. *n* = 5. R, rostral; C, caudal. Scale bar, 2 mm. **d** Axonal projection of M/T cells in the OC. M/T-cell axons in the OC sections of WT and ΔDV mice were visualized by fluorescence of YFP induced by the *Pcdh21* promoter. The following OC regions were examined: lateral olfactory tract, LOT; anterior olfactory nuclei, AON; piriform cortex, Pir; olfactory tubercle, OT; cortical amygdala, CoA; and medial amygdala, MeA. *n* = 5. Scale bars, 250 μm

We also analyzed secondary projection to the olfactory cortex in the absence of targeting of OSN axons (Fig. 3). M/T-cell axons were visualized by fluorescence of YFP induced by the M/T-cell specific Pcdh21 promoter (Fig. 3a). Various olfactory cortical regions (lateral olfactory tract, LOT; anterior olfactory nuclei, AON; piriform cortex, Pir; olfactory tubercle, OT; cortical amygdala, CoA; and medial amygdala, MeA) in the ∆D mutant were inspected for possible defects in M/T-cell projections using the WT sections as controls. Aside from LOT appearing thinner in the ∆D, no anatomical differences were noted between the ∆D and WT mice. However, the above observations do not necessarily conclude that the dorsal M/T cells in the ∆D mice project their axons normally to the olfactory cortex, as M/T cells project their axons also from the ventral-region olfactory bulb. We, therefore, inspected another mutant mouse, ∆DV^28^, in which glomeruli are entirely absent from both dorsal and ventral regions of the olfactory bulb. Using the *goofy* promoter activated in immature OSNs, OSNs were totally eliminated from the olfactory epithelium by activating the diphtheria-toxin gene from *NSE-STOP-DTA*^29^ using the *goofy-Cre* driver^30^ (Fig. 3a, b). Here, we obtained basically the same results with the ∆DV mice as those found in the ∆D mutant (Fig. 3c, d). One notable difference was that in addition to the LOT, signals were thinner in the Pir and MeA in the ∆DV. This is probably due to the absence of OSN inputs. These observations indicate that cortical projection of M/T cells likely occurs independent of synapse formation with OSN axons, at least for initial stages of circuit formation. Targeting of M/T axons appear to be hard-wired and instructed by the genetic program as seen in the fly^12^.

### Synaptic contacts in the absence of OSN activity

**Fig. 4:**
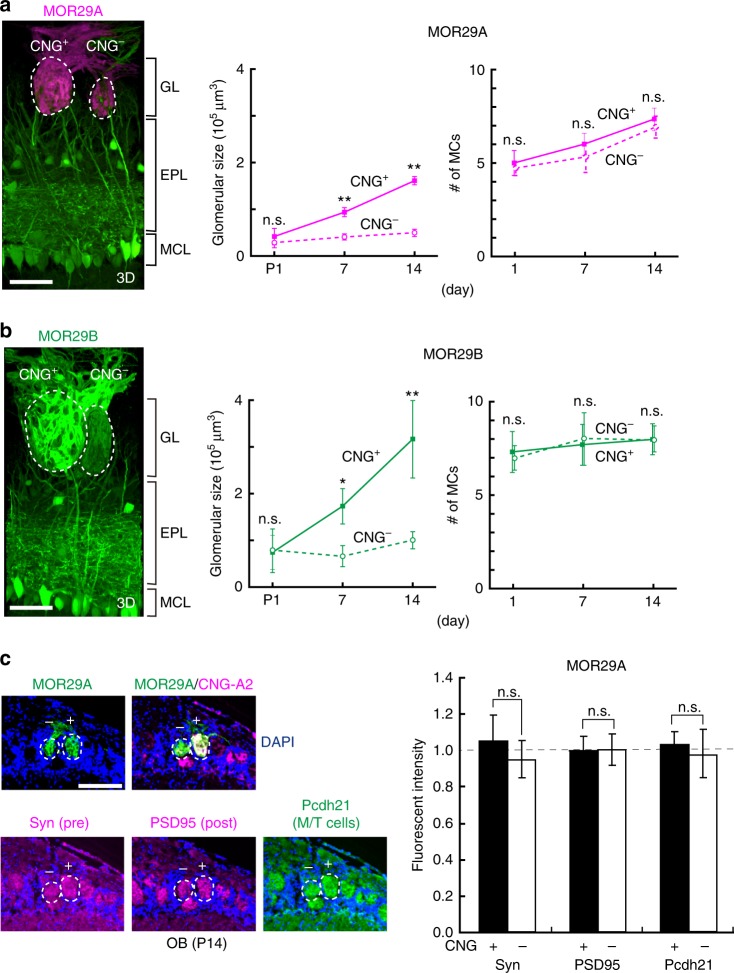
Matching of mitral cells (MCs) with the CNG^−^ glomeruli. **a** MOR29A glomeruli in the hemizygous female CNG-A2. Image: mitral-cell dendrites are compared between the CNG-positive (+) and -negative (–) glomeruli with the same odorant receptor identity, MOR29A. OSN axons of MOR29A glomeruli (magenta for CFP) and connecting MCs (green for YFP) were analyzed by two-photon laser microscopy. Three-dimensional videos are available in the supplementary information. GL, glomerular layer; EPL, external plexiform layer: MCL, mitral cell layer; FP, fluorescent protein. Scale bar, 100 μm. Graph: The glomerular sizes and numbers of connecting MCs are compared between the CNG^+^ and CNG^−^ glomeruli at P1, 7, and 14. *n* = 3 animals, *n* = 5–8 glomeruli for each time point. Error bar, S.E.; n.s., *p* > 0.05; *0.05 > *p* > 0.01; **p < 0.01; Welch’s *t*-test. **b** MOR29B glomeruli in the hemizygous female CNG-A2 KO. Dendrite connection and synapse formation of MCs were compared between the CNG^+^ and CNG^−^ glomeruli of MOR29B as shown for the MOR29A glomeruli in **a**. MOR29B glomeruli are shown in green, because they were labeled with YFP whose fluorescence cannot be distinguished from that of YFP for MCs. **c** Synapse formation in the CNG^+^ and CNG^−^ glomeruli. Olfactory bulb (OB) sections containing the MOR29A glomeruli at P14 were immunostained with antibodies against synaptic markers, Synaptophysin (Syn, pre-synaptic marker) and post-synaptic density 95 (PSD95, post-synaptic marker). Serial OB sections were stained with antibodies against CNG-A2 (CNG-channel marker) and Pcdh21 (M/T-cell marker). No differences were found in staining between the CNG^+^ and CNG^−^ glomeruli. Scale bar, 100 μm. Error bar, S.E.; n.s., *p* > 0.05; Welch’s *t*-test

Dendrite de-arborization generally occurs in response to neuronal activity of presynaptic cells^31^. In the mouse olfactory system, it has been reported that dendritic pruning of mitral cells is delayed when the external naris is occluded^32^ or the cyclic nucleotide-gated (CNG) channel is knocked out^33^. Interestingly, however, Ron Yu and his colleagues observed that Kir2.1-expressing mice where all the OSN activities were blocked, did not demonstrate any defects in dendrite selection of mitral cells^34^. In order to determine whether the OSN activity affects partner matching of mitral-cell dendrites, we examined the hemizygous female KO of CNG-A2^33^ (Fig. 4). We chose to use the CNG^+/−^ system, because it allows us to study the activity dependency focusing on a particular odorant receptor in two adjacent glomeruli, one is for the CNG^+^ OSNs and the other is for CNG^−^.

As the *CNG-A2* gene is located on the mouse X chromosome^35^, stochastic X-chromosome inactivation allows the mosaic analysis of CNG-channel KO^33,35,36^. Due to the differential expression of activity-dependent glomerular-segregation molecules^16,26^, each glomerulus is duplicated in the hemizygous KO of CNG-A2. For this experiment, we analyzed MOR29A and MOR29B glomeruli (Fig. 4). To visualize the mitral-cell dendrites, CNG-A2 KO was crossed with the Tg pThy1-YFP mouse. It was found that dendrite selection of mitral cells was markedly delayed as reported^33^. However, no differences were found in the number of contacting mitral cells between the CNG-positive and CNG-negative glomeruli, although the glomerular sizes were smaller for CNG^−^ glomeruli than for CNG^+^ (Fig. 4a, b, and Supplementary movies 6, 7). In our study, mitral cells did not demonstrate any notable preference in extending their dendrites to the CNG^+^ and CNG^−^ glomeruli even under the competitive situation. To examine the synapse formation in the CNG^+^ and CNG^−^ glomeruli for MOR29A, olfactory bulb sections were immunostained with antibodies against Synaptophysin (Syn, pre-synaptic marker) and post-synaptic density 95 (PSD95, post-synaptic marker). No differences were found in the staining of Syn or PSD95 between the CNG^+^ and CNG^−^ glomeruli (Fig. 4c).

**Fig. 5:**
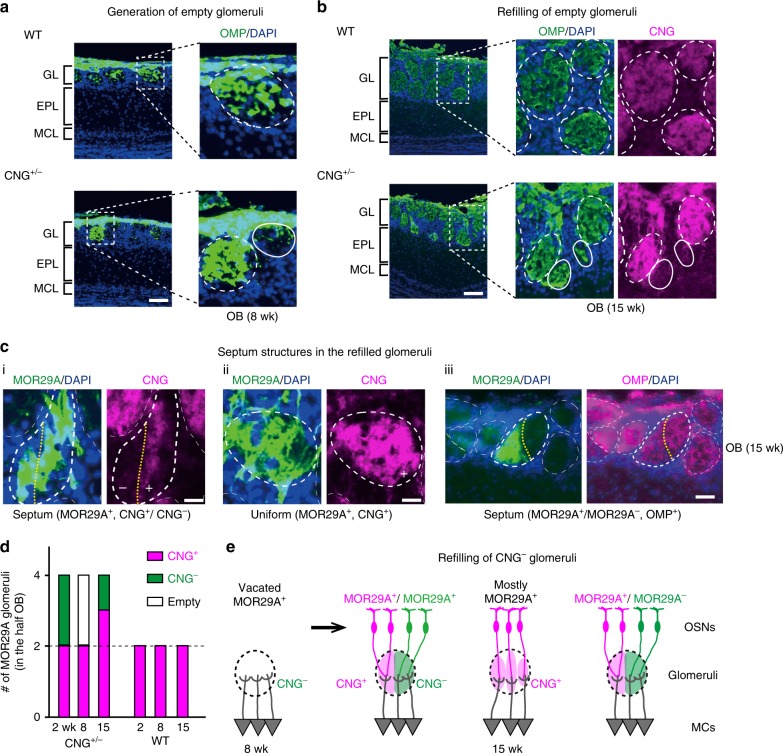
Activity-dependent degeneration and regeneration of synapses. **a** Generation of empty glomeruli. Coronal olfactory bulb (OB) sections (8 weeks) from the WT and CNG-A2^+/−^ female mice were stained with anti-OMP antibodies to detect OSN axons. Due to the competition in the hemizygous KO, CNG^−^ OSNs were eliminated and empty glomeruli (encircled) devoid of OSN axons were generated. Sections were also stained with DAPI. *n* = 5 animals. GL, glomerular layer; EPL, external plexiform layer; MCL, mitral cell layer. Scale bar, 50 μm. **b** Refilling of empty glomeruli. Coronal sections (15 weeks) of the WT and CNG-A2^+/−^ female mice were immunostained with antibodies against OMP and CNG-A2. Refilled glomeruli (CNG^−^) are encircled. CNG^+^ glomeruli are encircled by dotted lines. *n* = 5 animals. GL, glomerular layer; EPL, external plexiform layer; MCL, mitral cell layer. Scale bar, 50 μm. **c** Septum structures in the refilled glomeruli. Coronal sections of the Tg MOR29A mice (15 weeks) with the background of CNG-A2^+/−^ KO were immunostained with antibodies against GFP, CNG-A2 and OMP. Sections were also stained with DAPI. Glomerular structures are encircled. In the refilled MOR29A glomeruli, septum structures (dotted line) were often formed separating the CNG^+^ and CNG^−^ OSN axons of regenerated MOR29A^+^ OSNs (i). In some cases, the vacated CNG^−^ glomeruli were refilled with CNG^+^ axons, mostly MOR29A^+^, without forming a septum (ii), or with MOR29A and other odorant receptor species forming a septum (iii). **d** Rations of the CNG^+^ and CNG^−^ glomeruli. MOR29A glomeruli were analyzed at 2, 8, and 15 weeks. At 8 weeks, most CNG^−^ MOR29A glomeruli were devoid of OSN axons. **e** Schematic diagrams of degeneration and regeneration of CNG^+^ and CNG^−^ axons. CNG^−^ axons are eliminated due to the competition with CNG^+^ axons and the vacated CNG^−^ glomeruli are partly occupied by CNG^+^ OSN axons forming the septum structure within glomeruli. GL, glomerular layer; EPL, external plexiform layer; MCL, mitral cell layer. Scale bars, 50 μm

In the mouse olfactory system, OSNs are constantly regenerated in adult animal^37^. In contrast, mitral cells cannot be supplied after their initial migration from the subventricular zone in embryo^38^. As a result, all mitral-cell dendrites maintain their original existence throughout the life^27^. In the hemizygous female KO of CNG-A2, CNG^−^ OSNs are eventually eliminated in an activity-dependent manner under the competitive situation with the CNG^+^ glomeruli^35^. We studied what would happen to the vacated CNG^−^ glomeruli (Fig. 5). When olfactory bulb sections were analyzed in the 2 week-old hemizygous KO, both CNG^+^ and CNG^−^ glomeruli were equally found. However, at 8 week, we frequently found vacant glomeruli devoid of OSN axons, although the mitral-cell dendrites remained intact (Fig. 5a). In later stages, the vacant glomeruli were filled with newly projecting OSN axons, synapsing with the pre-existing dendrites of mitral cells (Fig. 5b). In order to precisely determine whether the odorant-receptor specificity is maintained after refilling in the glomeruli, we crossed the Tg mouse, containing *MOR29A-ires-gapECFP*, with the CNG-A2 hemizygous female KO. As mentioned earlier, duplicated structures were found for Tg MOR29A glomeruli at earlier stages (2 week): one was CNG^+^ and the other was CNG^−^. However, CFP-stained OSN axons ultimately disappeared from the CNG^−^ glomeruli. In the aged animal (15 week), CFP-signals returned to the vacated glomeruli, often forming septum structures that separate the CNG^+^ and CNG^−^ OSN axons of regenerated MOR29A^+^ OSNs (Fig. 5c, i). In some cases, the vacated CNG^−^ glomeruli were refilled with CNG^+^ axons, mostly MOR29A^+^, showing a sparse pattern of CFP staining rather than a uniform one without forming a septum (Fig. 5c, ii). In other cases, a septum was formed in the refilled glomeruli, separating the OMP^+^ region into two parts; one is CFP^+^ and the other is CFP^−^ (Fig. 5c, iii). The CFP^−^ region in the refilled MOR29A glomeruli is likely filled with OSN axons of odorant receptor species other than MOR29A. Partner matching was detected equally for both CFP^+^ and CFP^−^ areas within the refilled glomerular structure (Fig. 5c–e). These findings indicate that in the CNG^−^ vacated glomeruli, pre-existing mitral-cell dendrites are able to form synapses with newly projecting OSN axons likely possessing different odorant receptor identity from the original one.

### Glomerular structures in the OSN-ablated olfactory bulb

In the mouse olfactory system, the neural circuit between the olfactory epithelium and olfactory bulb can be reconstituted, as OSNs are constantly regenerated^39^. Furthermore, granule cells and periglomerular cells are continuously being supplied. In adult animals, non-functional glomeruli are eliminated under the competitive situation and replaced by other OSN axons^40^. This is in contrast to mitral cells that cannot be supplied after their initial migration from the subventricular zone to the embryonic olfactory bulb during early development^41^. Regeneration also occurs after the removal of OSN axons by chemical and physical ablations^42,43^. Although the glomerular map is reconstituted after ablation, the map appears to be imprecise, often generating ectopic glomeruli^42,43^.

**Fig. 6:**
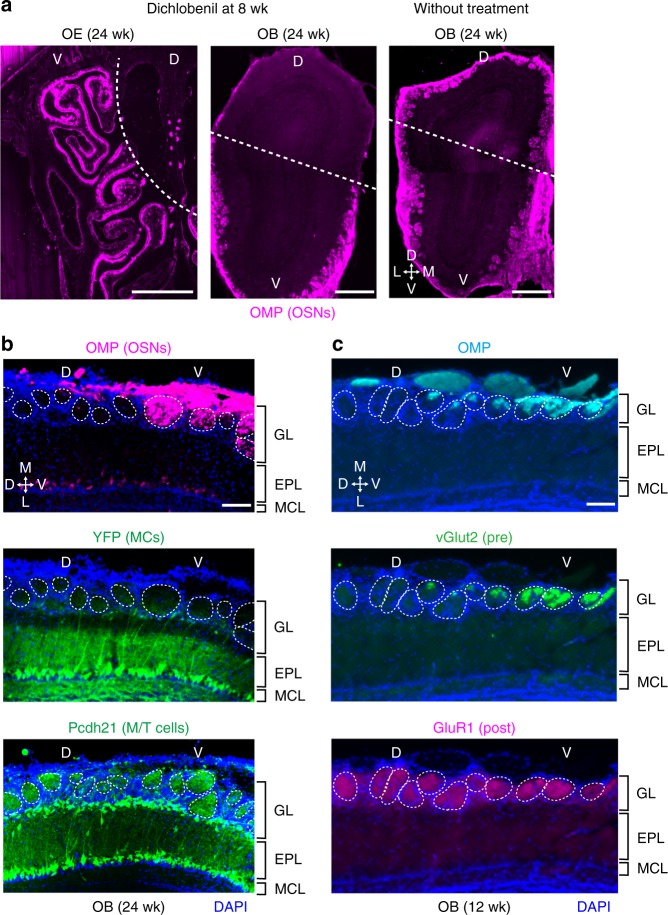
Ablation of D-region glomeruli. **a** Chemical ablation of OSNs in the D-zone olfactory epithelium (OE). Dichlobenil was intraperitoneally injected into the 8 week-old pThy1-YFP mice (10 mg/ml in DMSO, 2.5 μg/kg). OE sections were immunostained with anti-OMP antibodies (magenta). Sixteen weeks after the dichlobenil treatment, OSNs and OSN axons (OMP-positive) were completely absent from the D-zone OE and the D-region olfactory bulb (OB), respectively. *n* = 5 animals. Scale bars, 500 μm. Dotted lines indicate the borders of V and D regions. **b** OB sections of the dichlobenil-treated pThy1-YFP mice. The sections (24 weeks, 20 μm thick) were stained with antibodies against OMP (OSN axon marker) and Pcdh21 (M/T-cell marker). D-zone OSNs were totally removed by the chemical ablation. In contrast, post-synaptic glomerular structures were stably maintained even without the pre-synaptic OSN axons at least for 24 weeks, keeping the primary dendrites and their tuft structures intact. Note that in the dichlobenil-treated OE, OSNs do not regenerate, because their stem cells are also eliminated by the chemical ablation. *n* = 5 animals. GL, glomerular layer; EPL, external plexiform layer; MCL, mitral cell layer. Scale bar, 100 μm. **c** Synapse formation in the glomeruli near the D-V border. OB sections were analyzed for synaptic markers after 12 weeks of chemical ablation. On the dorsal side, glomeruli were stained for the post-synaptic marker, GluR1, but not for the pre-synaptic marker, vGlut2, although the ventral-side glomeruli were stained for both. *n* = 5 animals. GL, glomerular layer; EPL, external plexiform layer; MCL, mitral cell layer. Scale bar, 100 μm

In order to study how the glomerular structure is maintained after the forced removal of OSN axons, we ablated OSNs by an intraperitoneal injection of dichlobenil (2,6-dichlobenzonitrile) into adult pThy1-YFP mice at 8 weeks (Fig. 6). As reported^42^, dichlobenil treatment resulted in severe destruction of all OSNs in the dorsal olfactory epithelium, whereas the effects in the ventral olfactory epithelium were minor (Fig. 6a). Sixteen weeks after dichlobenil treatment, OSN-ablated glomeruli in the dorsal olfactory bulb still maintained the caged structure filled with periglomerular cells even in the absence of OSN axons (Fig. 6b). To examine the synapses in the vacant glomeruli after 12 weeks of chemical ablation, olfactory bulb sections were immunostained with antibodies against synapse markers, vGlut2 and GluR1 (Fig. 6c). On the dorsal side of the border, glomeruli were stained for the post-synaptic marker, GluR1, but not for the pre-synaptic marker, vGlut2, although the ventral-side glomeruli were stained for both. Mitral-cell dendrites may retain their connections to periglomerular cells, and there was no sign of elimination or reconnection of primary dendrites (Fig. 6b, c). It seems that once the glomerulus is set up with OSN axons, periglomerular cells, and primary dendrites of mitral cells, OSN axons can be removed and the rest of the structure remains at least in crude form. Since dichlobenil treatment eliminated not only the existing OSNs but also their stem cells, we did not observe refilling of the vacated glomeruli in the dorsal-region olfactory bulb. Interestingly, however, Brian Key and his colleagues reported that in their chemical ablation, ventral-zone OSNs expressing the P2 odorant receptor miss-targeted to the dorsal-region olfactory bulb^42^. This observation may indicate that empty dorsal-region glomeruli after the OSN ablation can be refilled by ventral-region axons without regard to their odorant-receptor specificity.

## Discussion

In the present study, we analyzed partner matching between the OSN axons and mitral-cell dendrites in the mouse olfactory system. The question to be answered was how both parties are able to find the right partners for correct synapse formation. Our present results indicate that mitral-cell dendrites find their partner OSN-axons based on their proximity to the target glomeruli without regard to odorant-receptor specificity, tending to connect to the nearby glomerulus. If the odorant receptor identity of glomeruli is not directly recognized by the mitral-cell dendrites for synapse formation, how is the odor information, detected in the olfactory epithelium, transmitted correctly to the olfactory cortex? In the mouse olfactory system, synapse formation in the olfactory bulb is flexible in terms of odorant-receptor specificity, although wiring beyond the mitral cells appears to be genetically programed. Our study indicates that matching occurs based on where the OSN axons converge and where the mitral cells are located after the migration, regardless of the odorant-receptor specificity of glomeruli. In other words, glomerular location in the olfactory bulb rather than the odorant-receptor specificity of glomeruli is important for the wiring with particular mitral cells. This leads to an intriguing possibility that odor quality is given by the location of glomeruli in the olfactory bulb rather than their odorant receptor identity and that the olfactory map contains distinct functional domains. We assume that glomeruli for particular odorant receptors could move around from one functional domain to another, aided by the fluctuation of expression levels of axon guidance molecules^12,13^. If dendrite selection takes place based on the proximity model, glomeruli for particular odorant receptors should be able to form functional circuits with mitral cells wherever they are located in the olfactory bulb. This flexibility may allow the mammalian olfactory system to adapt to the new odor environment and changes in the order quality during evolution.

It should be noted that mitral cells can survive without synapsing to OSN axons as reported for the fly^13^. In the ΔD and ΔDV mice, mitral cells survive and project their axons to the olfactory cortex without synapsing with OSN axons. How do they survive without receiving OSN activity? Activities from interneurons, e.g., periglomerular cells, may support mitral cells for survival even when the OSN activity is absent. It would be interesting to study how the mitral cells can be maintained without sensory stimulation from OSNs. Considering that mitral cells do not regenerate unlike OSNs, it may be unique characteristics of mitral cells to maintain the glomerular structure throughout the lifetime. It is interesting to ask whether the wiring of mitral cells with the specific cortical neurons could instruct the synapse formation with OSN axons, or inversely, the synapse formation with particular glomeruli would affect the cortical projection of mitral cells. Our studies with ∆DV mice indicate that mitral-cell projection to the olfactory cortex is not markedly affected by the ablation of glomerular formation, at least for the initial stages. This observation supports the idea that the cortical projection of mitral cells takes place independent of synapse formation with OSN axons, and that olfactory circuit is hard-wired beyond mitral cells as in the fly^12,13^.

Our study as well as previous works by others^42,43^ demonstrates that regenerated OSN axons in the adult have the capacity to grow back to the olfactory bulb and re-establish functional synapses with mitral cells following olfactory nerve injury. However, the projection patterns are markedly altered for both glomerular location and segregation. Our study demonstrates that glomerular structures on the mitral-cell side remain intact even after the total ablation of OSN axons. This has important implications for *Parosmia* in the human system. The olfactory dysfunction is caused by physical damage that destroys the wiring of OSNs from the olfactory epithelium to the olfactory bulb^44^. The dendritic stability of mitral cells and imprecise regeneration of the glomerular map is probably associated with *Parosmia*, which is characterized by the inability of the olfactory system to properly identify odor quality after the regeneration of the map.

Our present study strongly supports the proximity model for dendrite selection of mitral cells in the mouse olfactory system. This explains some curious observations for OSN projection and glomerular formation. For example, non-odorant receptors, such as β2-adrenergic receptor, or non-mouse odorant receptors like rat I7 receptor, are able to form their own glomeruli synapsed with mitral cells^45,46^. Furthermore, ectopic glomeruli often found in the KO mice for axon-guidance molecules also form synapses with mitral cells^12^. Since the glomerular positions and their partner mitral cells are not pre-assigned for the ectopic glomeruli, the proximity model explains these findings. It should be noted that ectopic glomeruli are gradually eliminated in an activity-dependent manner under the competitive situation^16,35^. Considering the stability of mitral-cell dendrites in glomeruli, the activity-dependent elimination/selection may be taking place at the level of OSN axons, but not mitral-cell dendrites. In the adult, the vacant olfactory bulb space after elimination of ectopic glomeruli is eventually taken over by functional OSN axons with different odorant receptor identities, keeping the pre-existing glomerular structure intact on the mitral-cell side. This observation is also consistent with the proximity model.

Our present study supports the proximity model whereby mitral cells tend to connect primary dendrites to the nearest neighboring glomeruli based on a possible requirement that each mitral cell can connect to one glomerulus. For mitral cells, pruning of lateral dendrites is another important issue. In our study, the selection of these secondary dendrites was not analyzed. However, we assume that lateral-dendrite selection also takes place restricted to their physical locations rather than to the specificity of the partner cells. Although the proximity model explains a number of puzzling observations in the wiring of mitral cells, it raises additional questions. Our present data indicate that dendrite selection and cortical projection of mitral cells may take place independent of each other during the earlier stage of circuit formation. Is basic wiring of mitral-cell axons to various regions in the olfactory cortex genetically-programmed? Is there any activity-dependent selection of mitral-cell circuits at the level of axons or their branches in the olfactory cortex? These interesting questions are to be clarified by the future experiments. It is certain that the mouse olfactory system will continue to serve as a powerful tool for the study of neural circuit formation in the mammalian sensory systems.

## Methods

### Animals

The pThy1-YFP transgenic (Tg) mice (line G) and CNG^−^A2 knockout (KO) mice were obtained from the Jackson Laboratory. The MOR29A/B BAC Tg mice^25^, ΔD^18^, and ΔDV^28^ mice were reported previously. The H-MOR29A Tg mice were generated according to the published procedure^23,24^. Mutant mice were crossed with MOR29A/B BAC Tg or pThy1-GFP Tg to visualize the MOR29A and MOR29B glomeruli with mitral cells, respectively. The pPcdh21-YFP Tg mice were generated by linking the *Pcdh21* promoter^19,47^ to the tau-YFP-pA sequence. All animal experiments were performed in accordance with the University regulations and guidelines, and approved by the institutional review committees of Graduate School of Science, University of Tokyo and School of Medical Sciences, University of Fukui.

### Immunohistochemistry

Embryonic and neonatal mice (≦PD7) were fixed in toto by immersion in Mildform 10 NM (Wako Pure Chemical). Grown-up mice (>PD7) were anesthetized with sodium pentobarbital (2.5 mg/animal) and perfused intracardially with Mildform 10 NM. Coronal cryostat sections (20 μm thick) of the olfactory bulb and olfactory epithelium were prepared according to the standard procedure^23^. To identify the MOR29A and MOR29B glomeruli in olfactory bulb sections, fluorescent microscope images of C/YFP were taken. The sections were activated by autoclaving (105 °C, 5 min) and blocked for 1 h at room temperature (RT) in PBS containing 0.1% Tween 20 and 5% skim milk and then incubated overnight with antibodies. Antibodies used in this study are as follows: rabbit anti-CNG-A2 antibodies (1:200, Alomone Labs); rabbit anti-PSD95 antibodies (1:1000, Cell Signaling); mouse anti-Synaptophysin (Syn) antibodies (1:1000, Millipore); goat anti-OMP antibodies (1:1000, Wako Pure Chemical); rabbit anti-NQO1 antibodies (1:300, Abcam); guinea pig anti-vGlut2 antibodies (1:300, Millipore); rabbit anti-GluR1 antibodies (1:300, Abcam); rabbit anti-GFP antibodies (1:300, Invitrogen); chicken anti-GFP antibodies (1:300, Abcam). A guinea pig anti-Pcdh21 antiserum (1:300) was generated according to the published procedure^26^. Sections were incubated for 1 h at RT in an Alexa Fluor-conjugated secondary antibodies (1:300, Invitrogen) containing 1 µg/ml DAPI.

### Measurement of fluorescent signals

For fluorescent signals of immunostaining, digital images were captures with a digital CCD camera, C4742-95-12ERG (Hamamatsu Photonics). Tone was reversed and monochrome image was used for measurement. To quantitate the staining level of each glomerulus, the mean pixel intensity within the region surrounded by the periglomerular cell nuclei was measured using Scion Image (Scion Corp.). Expression levels of Pcdh21, Syn, and PSD95 in the glomeruli, were measured as average fluorescent intensities in the glomeruli and used for the normalization.

### Two-photon laser microscope analysis

Mutant mice were crossed with pThy1-YFP mice to visualize the mitral cells. Coronal vibratome sections (500 μm thick) of the olfactory bulb were incubated with FocusClear (CelExplorer Labs) for 3 h and then mounted with MountClear (CelExplorer Labs). Mitral-cell dendrites were imaged by the two-photon laser microscope (Olympus) with 920 nm laser and 25× objective lens (Olympus). Three-dimensional binocular visions of stack images were reconstructed with ImageJ (NIH). The numbers and percentages of YFP positive mitral cells in the pThy1-YFP Tg mice were counted based on the dendrite morphology. Sizes of glomeruli were measured with the voxel count of the glomerular structures multiplied by the voxel size. CNG-A2 expression was determined by immunohistochemistry of resliced cryostat sections (16 μm thick).

### TUNEL (TdT-mediated dUTP nick end labeling) assay

To examine apoptosis, fragmented DNA was detected in the apoptotic cells. DNA fragmentation is a characteristic hallmark of apoptosis. Terminal deoxynucleotidyl transferase dUTP nick end labeling (TUNEL) is an established method for the detection of DNA fragments. In this experiment, sections were permeabilized with Neuropore (R&D Systems) for 75 min at RT, and TUNEL was performed at 37 °C according to the manufacturer’s protocol (R&D Systems).

### Statistical analyses

All statistical analyses were performed using Excel 2003 (Microsoft) with the Statcel2 add-on (OMS).

## Supplementary information

Supplementary Information

Description of Additional Supplementary Files

Supplementary Movie 1

Supplementary Movie 2

Supplementary Movie 3

Supplementary Movie 4

Supplementary Movie 5

Supplementary Movie 6

Supplementary Movie 7

## Data Availability

The supporting data in this study are available on request from the corresponding author.
