# Supplementary material for: Primary dendrites of mitral cells synapse unto neighboring glomeruli independent of their odorant receptor identity
Source: Commun Biol. 2019 Jan 8;2:14. doi: 10.1038/s42003-018-0252-y (PMC6325062; doi:10.1038/s42003-018-0252-y)
Supplement: Supplementary file 1 — Supplementary Information [file 42003_2018_252_MOESM1_ESM.pdf]

Supplementary Information

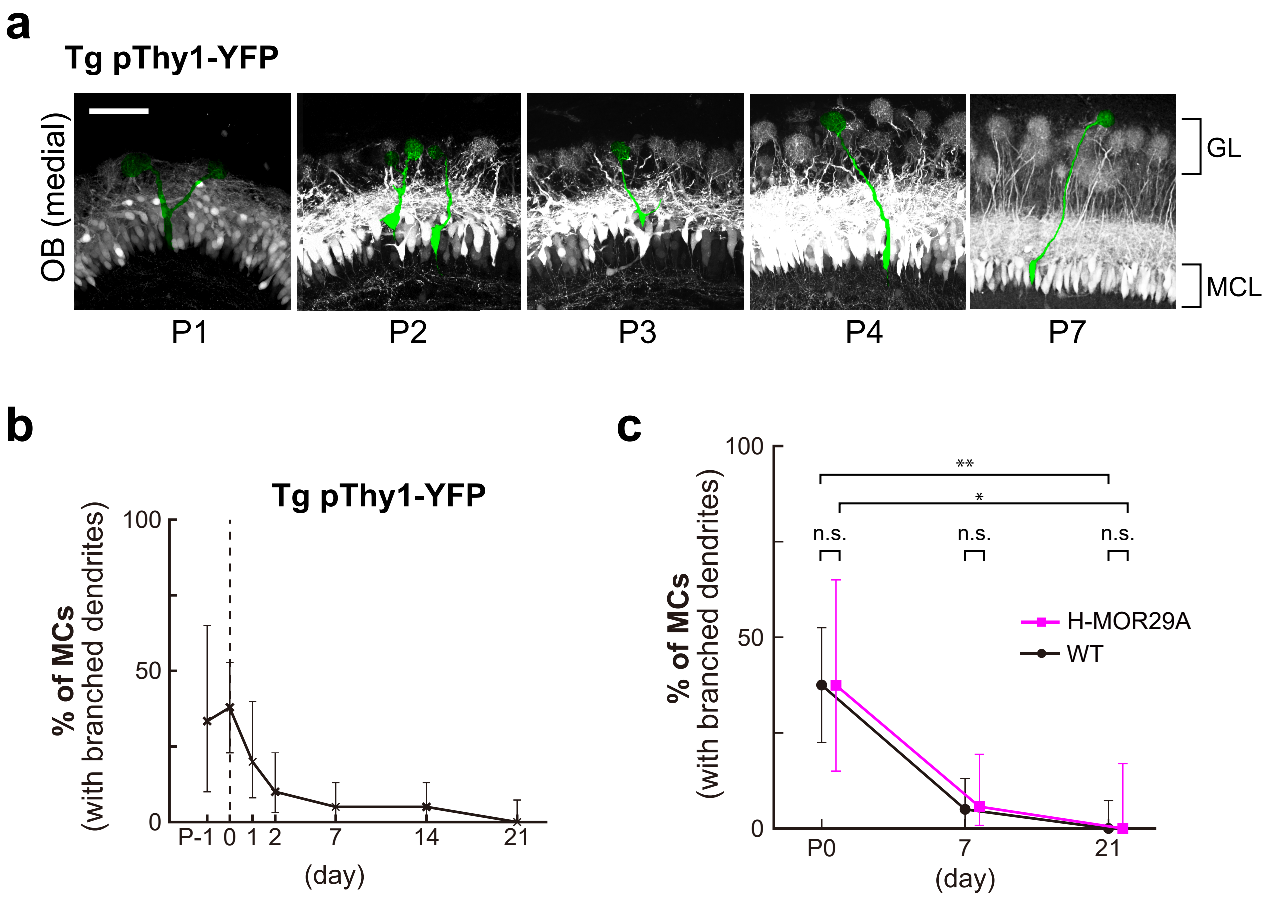

Supplementary Figure 1 Maturation of mitral-cell (MC) dendrites. (**a**) Selection of MC dendrites. Morphology of MC dendrites were analyzed in the Tg pThy1-YFP mice at P1, P2, P3, P4, and P7 by two-photon laser microscopy. MCs are visualized by YFP in the Tg Thy1-YFP mouse. Typical MCs are colored in green. GL, glomerular layer; MCL, mitral cell layer. Scale bar, 100 µm. (**b**) Dendrite selection of MCs. Ratios (%) of MCs with branched dendrites are plotted at P-1, P0, P1, P2, P7, P14, and P21. $n = 3$ animals. Error bar, S.E. (**c**) Dendrite maturation of MCs in Tg H-MOR29A mice. Ratios (%) of MCs with branched dendrites were measured plotted at P0, P7, and P21. $n = 3$ animals. Error bar, S.E.; n.s., $p > 0.05$; *, $0.05 > p > 0.01$; **, $p < 0.01$; Welch's *t*-test.

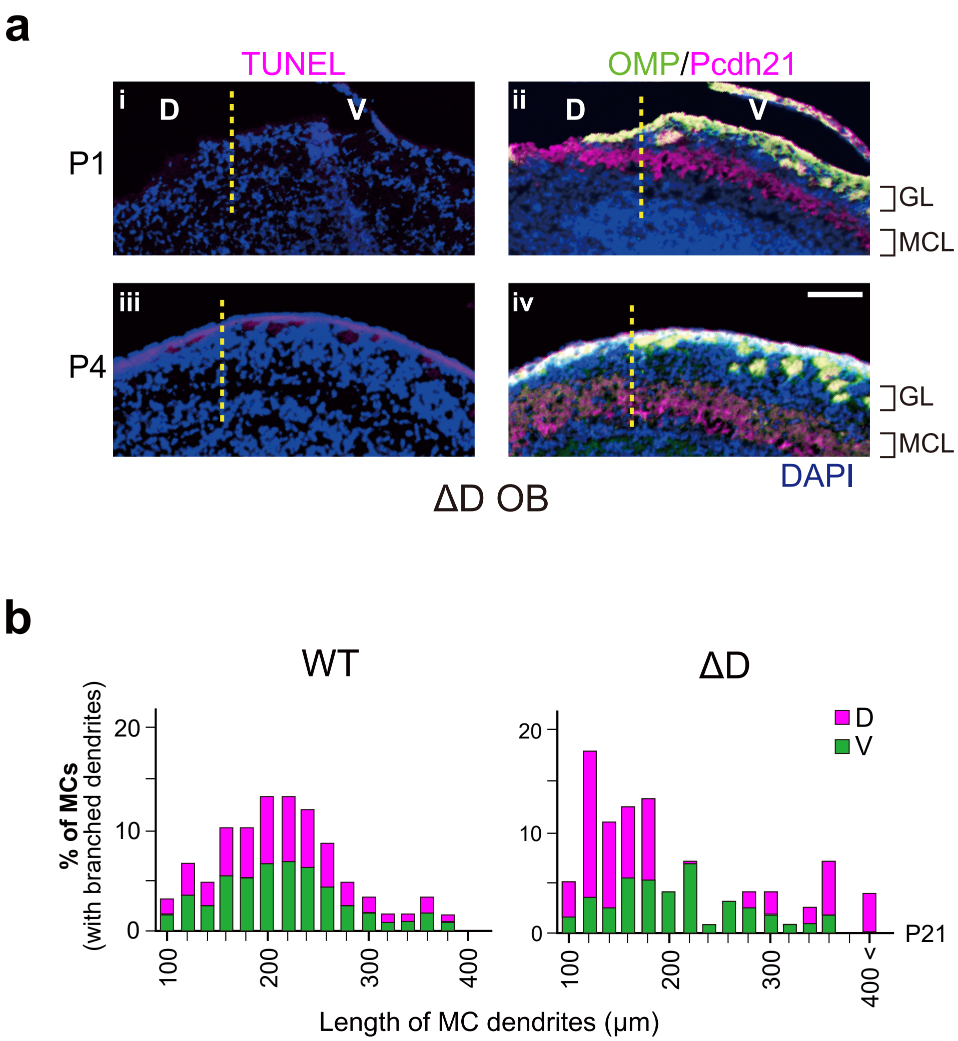

Supplementary Figure 2 Mitral cells (MCs) and their dendrites in the ΔD mice. (**a**) TUNEL assay for MCs. To examine the apoptosis of MCs in the ΔD mice, OB sections were analyzed at P1 and P4 by the TUNEL assay (see Methods) that detects fragmented DNA in apoptotic cells. Labeled signals (magenta) in the TUNEL assay were rarely found in the ΔD OB (i, iii). Serial OB sections were immunostained with antibodies against OMP (green) and Pcdh21 (magenta) (ii, iv). *n* = 3 animals. D, dorsal; V, ventral; MCL, mitral cell layer. Scale bar, 100 μm. (**b**) Detection of MCs with long primary dendrites crossing over the V-D border. Lengths of primary dendrites were individually measured for each MC near the D-V border in the WT and ΔD mice at P21. In the WT, the average length of primary dendrites was 225 ± 9 μm. In ΔD, shorter dendrites (< 200 μm) were found in the D region OB, and longer dendrites (> 340 μm) were found in the V region OB. Fractions of MCs on the dorsal and ventral sides are shown by magenta and green bars, respectively. *n* = 3 animals.
