# Supplementary material for: Primary dendrites of mitral cells synapse unto neighboring glomeruli independent of their odorant receptor identity
Source: Commun Biol. 2019 Jan 8;2:14. doi: 10.1038/s42003-018-0252-y (PMC6325062; doi:10.1038/s42003-018-0252-y)
Supplement: Supplementary file 2 — Description of Additional Supplementary Files [file 42003_2018_252_MOESM2_ESM.pdf]

Description of Additional Supplementary Files

File Name: Supplementary Movie 1

Description: MOR29A glomeruli at P0. Three-dimensional (3D) images were analyzed by two-photon laser microscope for MC-dendrite maturation. Using the Tg pThy1-YFP mice crossed with Tg MOR29A, MCs and MOR29A glomeruli were analyzed at P0. OSN axons of MOR29A and MCs are colored in yellow and green, respectively. OB sections are 500 μm thick.

File Name: Supplementary Movie 2

Description: MOR29A glomeruli at P4. Three-dimensional images of MOR29A glomeruli were analyzed by two-photon laser microscope at P4. OSN axons of MOR29A and MCs are colored in red and green, respectively. OB sections are 500 μm thick.

File Name: Supplementary Movie 3

Description: MOR29A glomeruli at P14. Three-dimensional images of MOR29A glomeruli were analyzed by two-photon laser microscope at P14. OSN axons of MOR29A and MCs are colored in red and green, respectively. OB sections are 500 μm thick.

File Name: Supplementary Movie 4

Description: MOR29A glomeruli at P14. Three-dimensional images of MOR29A glomeruli were analyzed by two-photon laser microscope at P14. OSN axons of MOR29A and MCs are colored in red and green, respectively. OB sections are 500 μm thick.

File Name: Supplementary Movie 5

Description: H-MOR29A glomeruli at P21. Three-dimensional images of MOR29A glomeruli were analyzed by two-photon laser microscope at P21. OSN axons of MOR29A and MCs are colored in red and green, respectively. OB sections are 500 μm thick.

File Name: Supplementary Movie 6

Description: MOR29A glomeruli in the CNG hemizygous KO mice. Three-dimensional images of the duplicated MOR29A glomeruli were analyzed by two-photon laser microscope. OB sections (500 μm thick) of the CNG-A2$^{+/-}$ female were analyzed at P14. OSN axons of MOR29A and MCs are colored in red and green, respectively.

File Name: Supplementary Movie 7

Description: MOR29B glomeruli in the CNG hemizygous KO mice. Three-dimensional images of the duplicated MOR29B glomeruli were analyzed by two-photon laser microscope. OB

sections (500 µm thick) of the CNG-A2$^{+/-}$ female were analyzed at P14. Both OSN axons of MOR29B and MCs are colored in green.
